# Corrosion Inhibition of Carbon Steel in Neutral Chloride Solutions Using Salts of Primary Bile Acids

**DOI:** 10.1021/acsomega.4c06362

**Published:** 2024-09-18

**Authors:** Juan P. Aguilar-Barrientos, Máximo A. Pech-Canul, María A. Fernández-Herrera

**Affiliations:** Departamento de Física Aplicada, Centro de Investigación y de Estudios Avanzados del Instituto Politécnico Nacional, Unidad Mérida. Km. 6 Antigua Carretera a Progreso, Apdo. Postal 73, Cordemex, Merida, Yucatan 97310, Mexico

## Abstract

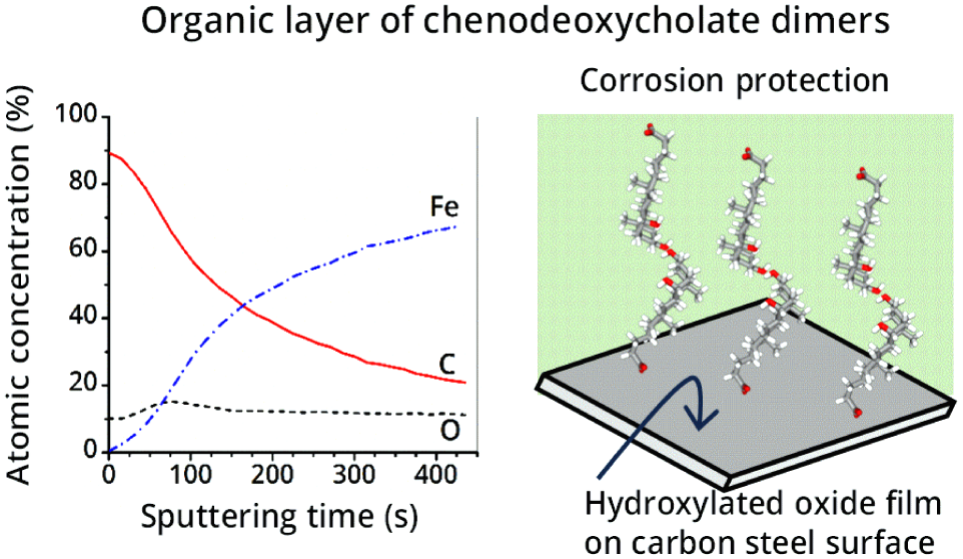

Due to growing environmental concerns and regulatory pressures, the demand for environmentally friendly corrosion inhibitors has increased. Biosurfactants are biodegradable and have a low toxicity. However, very few studies have reported on their potential use as corrosion inhibitors. The present study reports the novel application of two bile salts (sodium cholate NaC and sodium chenodeoxycholate NaCDC) as environmentally friendly corrosion inhibitors for carbon steel in a neutral 20 mM NaCl solution. The results of potentiodynamic polarization and electrochemical impedance measurements showed that when added at a concentration of 5 mM, the corrosion inhibition efficiencies of NaC and NaCDC were about 60% and 85%, respectively. The poor inhibitory character of NaC was confirmed by XPS analysis, revealing the formation of oxidative corrosion products on the steel surface. For the steel sample immersed in the solution containing NaCDC, the XPS measurements showed clear evidence of the presence of an organic layer and a passive oxide film on the steel surface. While the steroidal skeleton of NaC is characterized by marked biplanarity (considering its hydrophobic and hydrophilic faces), NaCDC features a steroidal ring with a hydrophilic edge (it does not exhibit biplanarity). Thus, the self-assembly and adsorption behavior of these bile salts on the steel surface are different, leading in the case of NaCDC to form a densely packed protective organic layer.

## Introduction

The cooling water system is a major auxiliary supporting service system in various industries including chemical, petrochemical, and power plants.^1^ A key factor to consider is the corrosivity of water, which depends on several physicochemical parameters, such as the chemical composition, pH value, temperature, and hydrodynamic conditions. Of particular concern is the presence of chloride ions, typically ranging from 150 to 500 mg/L in cooling waters,^2^ which can cause pitting corrosion in carbon steel, the primary material used in fabricating these cooling systems. An effective way to mitigate metal corrosion in cooling systems is by adding corrosion inhibitors.^3^ Corrosion inhibition involves introducing a chemical (the corrosion inhibitor) in small quantities to enhance the metal’s corrosion resistance. This method is especially effective in solutions with a pH of around 7 to 8, typical of cooling waters,^2^ and can be achieved with compounds capable of forming or stabilizing protective surface films.^4,5^ Representative compounds proven effective for inhibiting carbon steel corrosion in neutral solutions include chromates, nitrites, phosphates, borates, and benzoates.^6^ Although some of these chemicals have excellent corrosion inhibition performance, there are concerns regarding their toxic effects (as in the case of chromates). Thus, there is growing interest in developing environmentally friendly and cost-effective corrosion inhibitors. Among the proposed alternatives are organic compounds such as carboxylates,^7−9^ phosphonates,^10^ polyphenols, and other natural products.^11^

It is known that in neutral solutions, iron or mild steel corrosion begins at flaws located in the native oxide film.^12^ As recently reviewed by Godínez-Alvarez et al.^13^ and Kuznetsov,^9^ the corrosion inhibition mechanism of carboxylates for ferrous metals is mainly based on repairing such flaws according to pore plugging. Fe(II) ions produced in the local anodic zones become oxidized by dissolved oxygen to form Fe(III), and corrosion is suppressed by plugging the pores with weakly soluble Fe(III) compounds. The inhibitor’s adsorption plays a crucial role in the corrosion inhibition mechanism. For instance, Terryn and collaborators^14,15^ used XPS and FTIR-RAS to investigate the number of adsorbed molecules and the interaction mechanism between succinic acid and an oxide-coated iron surface. It was observed that succinic acid chemisorption occurred via deprotonation of the carboxylic acid group and formation of coordinatively bonded carboxylate species.

In addition, surfactants are amphiphilic molecules composed of a polar hydrophilic group attached to a nonpolar hydrophobic group (usually represented by a “head” and “tail”, respectively). In solution, they tend to form aggregates called micelles where the hydrophobic tails cluster together to form the core, while the hydrophilic heads remain in contact with water. The lowest concentration of surfactants at which micelles begin to form is referred to as the “critical micelle concentration” (CMC).^16^ Their amphiphilic nature also creates an affinity for adsorption at metal–metal oxide-solution interfaces; therefore, they are commonly used as corrosion inhibitors to protect metallic materials against corrosion. Recent reviews indicate^16,17^ that they offer advantages over traditional corrosion inhibitors due to ease of manufacture, reasonable cost, and low toxicity. The most widespread application of surfactants as corrosion inhibitors is in acidic media (corresponding to industrial processes such as acid steel pickling, chemical cleaning and treatment, oil well acidization, etc.). There are reports where surfactants such as long-chain quaternary ammonium bromides^18,19^ or glucosamide^20^ are used as corrosion inhibitors for mild steel in neutral chloride solutions. Gemini surfactants (a class with two hydrophobic chains and two hydrophilic heads, linked by a spacer at or near the head groups) have also been shown to be effective corrosion inhibitors of mild steel in aqueous solutions. Zhuang et al.^21^ tested four gemini imidazoline surfactants for the corrosion inhibition of X70 carbon steel in NaCl solutions in the pH range 5–9. Their electrochemical measurement results showed that the shorter the carbon chain length, the higher the inhibition efficiency, and the corrosion inhibition was better in alkaline solution than in neutral solution.

Biosurfactants are surface-active amphiphilic molecules produced by animals, plants, and microorganisms.^22^ Due to their outstanding physicochemical and biological properties, they find applications in several industries (environmental, cosmetic, medicinal, pharmaceutical, agricultural, etc.).^22,23^ Compared with synthetic surfactants, biosurfactants are biodegradable and have low toxicity. Thus, in principle, they should be considered potential corrosion inhibitors; however, as indicated in a recent review by Zehra et al.,^23^ only a few studies have attempted to use biosurfactants as corrosion inhibitors. Fawzy et al.^24^ synthesized two amino-based biosurfactants and showed that they worked as suitable corrosion inhibitors for mild steel in NaCl solutions. Helbert et al.^25^ showed that rhamnolipids derived from *Pseudomonas aeruginosa* exhibited a good corrosion inhibitory effect for carbon steel rebar in a simulated concrete pore solution.

**Figure 1:**
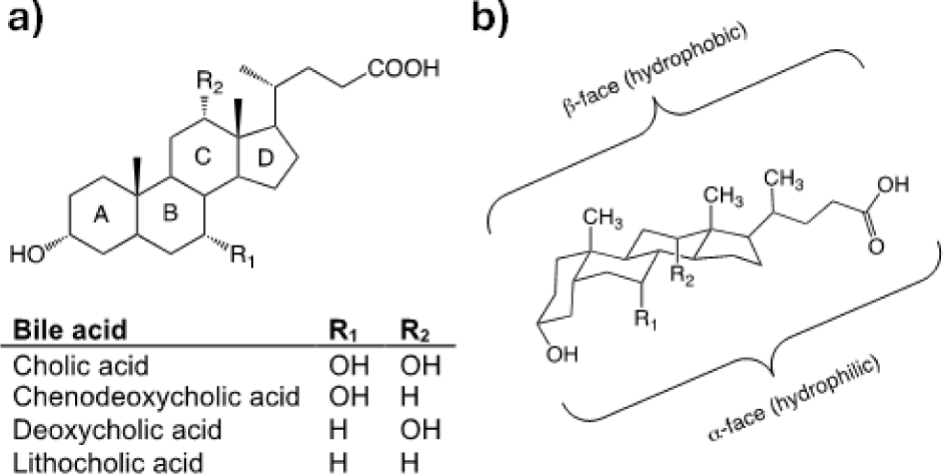
Planar (a) and chair (b) representations of the general molecular structures of primary and secondary bile acids. Letters A–D and labels R indicate the rings of the steroid skeleton and the positions of hydroxyl groups, respectively.

Bile acids (BAs) and bile salts (BSs) are physiologically essential biosurfactants synthesized in mammals’ livers and circulate through the digestive system.^26^ The primary BAs synthesized from cholesterol in the liver are cholic acid and chenodeoxycholic acid. In contrast, colon bacteria in the intestine convert secondary BAs (predominantly deoxycholic and lithocholic acids) from primary BAs. Naturally occurring BAs are hydroxylated derivatives of cholanic acid, a 24-carbon steroid, derived from a saturated tetracyclic hydrocarbon perhydrocyclopentanophenanthrene system, known as the steroid nucleus, which consists of three six-membered rings (A, B, and C), a five-membered ring (D), and a five-carbon side chain with a carboxylic group at the C-24 position (see Figure 1a). In BAs and BSs, the A and B rings bear a *cis*-fused configuration, inducing an overall bent shape, delineating a concave and convex side of the steroidal backbone. The convex β-face contains two methyl groups and behaves as a nonpolar, hydrophobic face, while the concave α-face acts as a hydrophilic face as it includes two or three polar hydroxyl groups (Figure 1b). Therefore, BAs and BSs are facially amphiphilic. Such a unique disposition of polar and nonpolar domains, along with the protonation of the carboxylic acid group on the lateral aliphatic chain, allows these molecules to be used in various applications.^27^ BSs possess strong dispersive power (which facilitates the stability of colloidal suspensions); therefore, they have attracted attention in the field of materials science for their use as dispersing agents in electrodeposition processes.^28,29^ Zhitomirsky and collaborators used salts of cholic acid, chenodeoxycholic acid, and deoxycholic acid as dispersing agents for the electrophoretic deposition of carbon (diamond, carbon dots, and graphene) or polytetrafluoroethylene (PFTE) materials on the type 304 stainless steel substrate.^30−32^ The electrochemical measurements showed that the advanced coating thus produced significant corrosion protection of stainless steel in a 3% NaCl solution.

To the best of our knowledge, the possible use of bile salts as corrosion inhibitors of reactive metals in aqueous solutions has not yet been reported. Thus, in this work, the inhibitory performance of salts of primary bile acids (cholic and chenodeoxycholic) for the corrosion of carbon steel in an aerated neutral solution containing 20 mM NaCl was investigated using conventional electrochemical measurements. This concentration is representative of a chloride limit of ∼500 ppm in cooling water^2^ (around 825 mg/L as NaCl). Bile salts were used rather than bile acids since salts exhibit high aqueous solubility.^26,33^ Due to their amphiphilic nature, bile salts tend to form aggregates in water through a self-assembly mechanism. Such a spontaneous organization of the molecules into well-defined and stable arrangements is driven by both the hydrophobic effect and hydrogen bonding (related to the positions and orientations of the hydroxyl groups).^34^ Bile salts also exhibit anionic properties attributed to the carboxylate group at the side chain. The latter leads to electrostatic interactions among the bile salt molecules and possible chemisorption on the metal surface. While sodium cholate (NaC) contains three hydroxyl groups, sodium chenodeoxycholate (NaCDC) contains only two. Such structural differences between these two molecules will affect their self-assembly and adsorption behavior on the carbon steel surface. So, to elucidate the mechanism of corrosion inhibition by these two bile salts, the chemical state and in-depth distribution of the composition of the surface films were investigated by X-ray photoelectron spectroscopy (XPS).

## Experimental Section

### Materials and Solutions

5 mm thick disc samples were cut from a commercial 1018 carbon steel rod, having the chemical composition (wt %) of Fe-98 703%, C-0,177%, Mn-0,636%, Cu-0,197%, Si-0,0578%, P-0,041%, and traces of some minor elements. Samples for electrochemical measurements were embedded in epoxy resin, leaving an exposed area of 1 cm^2^. Before each experiment, they were abraded with SiC papers up to 1200 grit, rinsed with distilled water, dried in a stream of hot air, and finally kept for 24 h at room temperature in a container with 45% RH to develop a native oxide film under controlled conditions.^35^

Sodium salts of cholic acid and chenodeoxycholic acid (Sigma-Aldrich) were obtained by neutralization of an ethanolic solution with NaOH (see the detailed procedure below).

The corrosive solution (blank) was prepared by dissolving reagent-grade NaCl in distilled water to a concentration of 20 mM, which approximates the chloride limit of 500 ppm in cooling water.^2^ The pH was adjusted to 7.0, aligning with the typical range for cooling water. For inhibited solutions, the corresponding amount of bile salt was added to the blank, to achieve a concentration of 5 mM. This concentration falls within the typical experimental CMC range for NaC and NaCDC^34^ and was selected to promote the formation of micelles with low aggregation numbers. The corrosion inhibition performance at higher concentrations, involving the formation of large micelles, will be evaluated in a future study. In each experiment, the solution was air-bubbled for 15 min before immersing the working electrode.

### General Procedure for the Synthesis of NaC and NaCDC

In a round-bottom flask, 0.25 mmol of the bile acid was placed and dissolved in 50 mL of ethanol. Subsequently, 0.5 mmol of NaOH was gradually added, and the mixture was heated to 60 °C until complete dissolution of NaOH was observed. The reaction mixture was stirred for 30 min and then cooled, resulting in the formation of a white precipitate with quantitative yields. The precipitate was filtered and recrystallized to ensure its purity.

### Characterization of NaC and NaCDC

The yielded sodium salts NaC and NaCDC were characterized by FTIR and RMN. Infrared spectroscopy (IR) was recorded on an Agilent Cary 630 Fourier Transform Infrared Spectroscopy (FTIR) spectrometer (range: 4000–600 cm^–1^) using an ATR interface. Processing of the spectra was performed by using MicroLab and ResPro software. The nuclear magnetic resonance ^1^H and ^13^C NMR were recorded in DMSO on an Agilent DD2 600 spectrometer at 600 and 150 MHz, respectively. The chemical shifts are reported in parts per million relative to residual solvent. Processing of the spectra was performed by using MestReNova software.

### Electrochemical Measurements

These were carried out in a three-electrode cell (with a Ag/AgCl reference electrode and a platinum foil as a counter electrode) using a Gamry series G300 potentiostat-galvanostat. The steel working electrode was immersed under open-circuit conditions in the corresponding solution for 2 h, enough time to stabilize the open-circuit potential (*E*_oc_). Then, the electrochemical impedance spectroscopy (EIS) measurement was performed under potentiostatic conditions at *E*_oc_ using a sine wave of 10 mV amplitude in a frequency range from 10 kHz to 10 mHz with 5 points/decade. This was followed by a potentiodynamic polarization test, starting 300 mV below *E*_oc_ and ending at 300 mV above it at a scan rate of 1 mV/s. Each experiment was conducted a minimum of three times to ensure the reproducibility of the results.

### Surface Analysis

XPS was used to analyze the chemical composition of films formed on the carbon steel surface after 2 h of immersion in the blank solution (20 mM NaCl, pH 7) without and with additions of NaC and NaCDC at a concentration of 5 mM. After the immersion test, the samples were dried, stored in a desiccator, and transferred to the XPS instrument within 3 h. XPS measurements were performed on a Thermo Scientific K-Alpha XPS spectrophotometer. A monochromatic Kα X-ray source was used with a spot area of 400 μm. To obtain an in-depth analysis of compositional information, two methods were used: (a) angle-resolved XPS, where the photoelectron takeoff angle (θ) was varied between 0° and 70° (with respect to the normal) and (b) XPS depth profiling, where sputtering was carried out with a 1 kV argon ion (Ar^+^) beam. In both methods, high-resolution core-level spectra were acquired for Fe 2p, O 1s, and C 1s. Binding energies (BEs) of the peaks in all the spectra were calibrated to the C 1s signal at 284.7 eV. The XPSPeak 4.1 program and the Shirley method for baseline extraction were used in the deconvolution and curve fitting of all the spectra.

## Results and Discussion

### Synthesis of NaC and NaCDC

The conversion of carboxylic acids to sodium carboxylates involves an acid–base neutralization reaction. The synthesis mechanism can be described as follows: The process begins with a carboxylic acid, which has the general formula R-COOH, where R represents the cholane skeleton and −COOH is the carboxyl group. The base used in this reaction is NaOH, a strong base that completely dissociates in water or alcohols to form sodium ions (Na^+^) and hydroxide ions (OH^–^). The carboxylic acid donates a proton (H^+^) to the hydroxide ion, resulting in the formation of water and a carboxylate ion (R-COO^–^). The carboxylate ion then associates with the sodium ion (Na^+^) from dissociated NaOH, leading to an ionic bond that produces sodium carboxylate (R-COONa).

### FTIR Characterization of NaC and NaCDC

**Figure 2:**
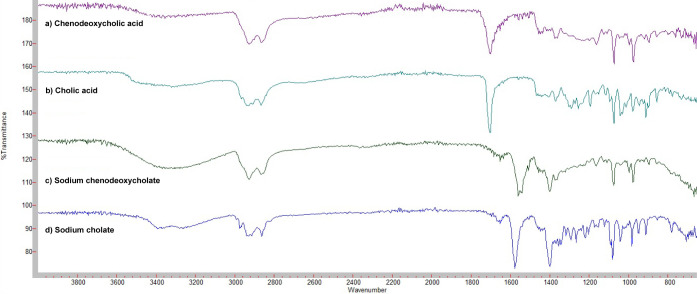
Infrared spectroscopy (IR) spectrum of bile acids (a,b) and their corresponding sodium salts (c,d), registered in a range of 600 to 4000 cm ^–1^.

Figure 2 shows the IR spectra of chenodeoxycholic and cholic acids followed by their corresponding sodium salts, chenodeoxycholate and cholate. The spectra of the acids show high similarity to each other, as do the spectra of the sodium salts, which supports the formation of the latter. In the region from 3400 to 3200 cm^–1^, broad bands corresponding to the vibrations of the intermolecular hydrogen-bonded O–H stretch bonds are observed. At 2900 cm^–1^, the stretching and bending bands are found, corresponding to the vibration of the C–H bonds and at 1450 cm^–1^, the scissoring bands corresponding to the CH_2_ groups, the most abundant in these structures. The region ranging from 1350 to 1150 cm^–1^ corresponds to less intense bands belonging to methylene twisting and wagging vibrations. The intense bands toward 1700 cm^–1^, observed for the bile acids, indicate the presence of the carbonyl group and correspond to the C–O stretching. The carboxylate ions give rise to two bands: a strong asymmetrical stretching band near 1580 cm^–1^ and a weaker, symmetrical stretching band near 1400 cm^–1^. The conversion of the carboxylic acids to the corresponding salt is confirmed by this couple of bands.^36^

### NMR Characterization of NaC and NaCDC

**Figure 3:**
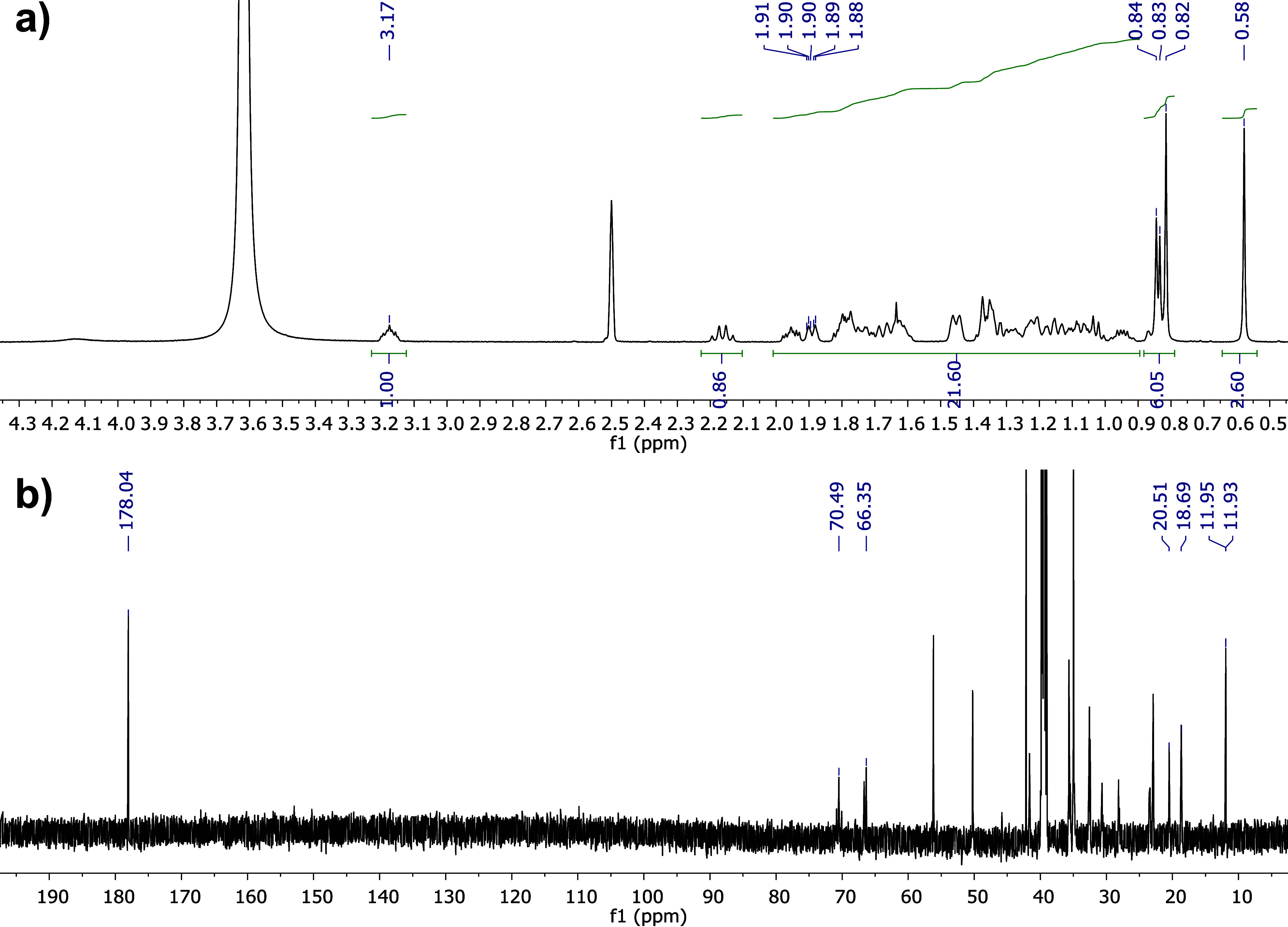
^1^H NMR (a) and ^13^C NMR (b) spectra of NaCDC.

In the ^1^H NMR spectrum of NaCDC (Figure 3a), a multiplet corresponding to H-3 as an OH base proton and integrating for a single signal is observed at 3.17 ppm. Proton H-12 is masked by the water signal found at 3.65 ppm, due to the high hygroscopicity of DMSO; however, the signal is confirmed in ^13^C NMR. The signals of methyls 18, 19, and 21, characteristic of the skeleton, are observed at 0.82, 0.58, and 0.84 ppm, respectively. The signal of methyl 21 appears as a doublet with a coupling constant of 6.55 Hz. For the ^13^C NMR spectrum of NaCDC (Figure 3b), the signal corresponding to the carboxylate is observed at 178.0 ppm. This is a key signal for the yield of the carboxylate anion as in the carboxylic acids, this peak appears around 187 ppm.^37^ At 70.5 and 66.4 ppm, the oxygen base carbons can be observed corresponding to the hydroxylated positions 3 and 7, respectively. The signals of the angular methyl carbons 18 and 19 and methyl 21 are observed at 11.95, 11.93, and 18.69 ppm, respectively.

**Figure 4:**
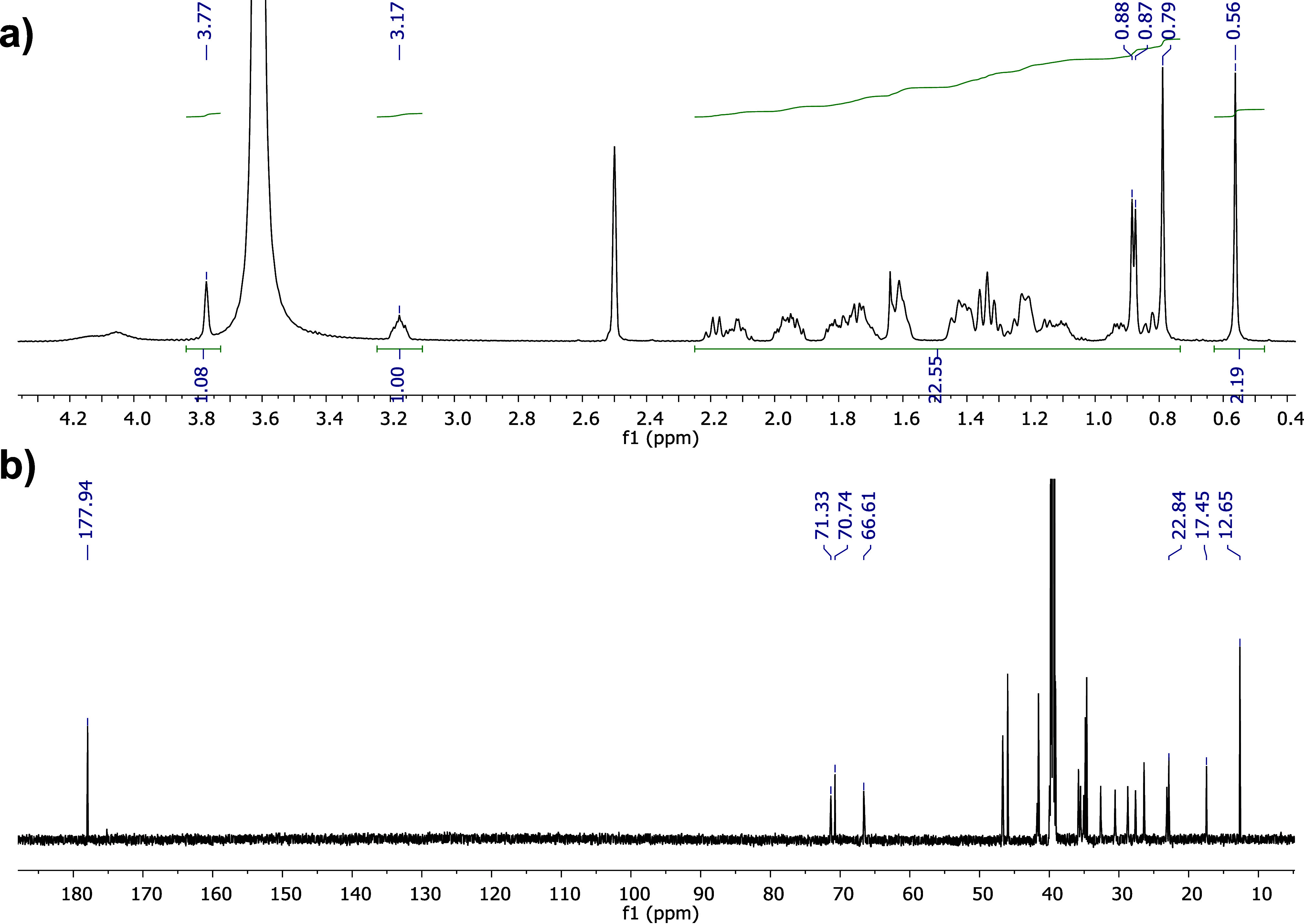
^1^H NMR (a) and ^13^C NMR (b) spectra of NaC.

Figure 4a shows the ^1^H NMR spectrum of NaC. At 3.77 ppm is the signal corresponding to H-12, followed by the multiplet at 3.17 ppm corresponding to H-3. As in Figure 3a, the H-7 signal is masked by the intense water signal observed around 3.6 ppm due to the high hygroscopicity of DMSO; however, the peak is confirmed in ^13^C NMR. The signals of the angular methyl 18 and 19 are seen as singlets at 0.79 and 0.56 ppm, respectively. The doublet observed at 0.87 ppm corresponds to methyl 21 and exhibits a coupling constant of 6.51 Hz. In the ^13^C NMR spectrum (Figure 4b), the characteristic signal of the carboxylate is observed at 177.9 ppm, confirming the structure.^37^ At 71.3, 70.7, and 66.6 ppm, the signals corresponding to the base protons of hydroxyl at positions 12, 3, and 7, respectively, are observed. Signals for methyls 18, 19, and 21 are observed at 17.5, 12.7, and 22.8, respectively. In all ^13^C NMR spectra, it was corroborated that the number of peaks coincided with the number of carbons for each structure to verify its purity. Furthermore, the melting points of both salts were determined at 288 °C (dec) for NaC and 320 °C (dec) for NaCDC.

### Electrochemical Characterization

**Figure 5:**
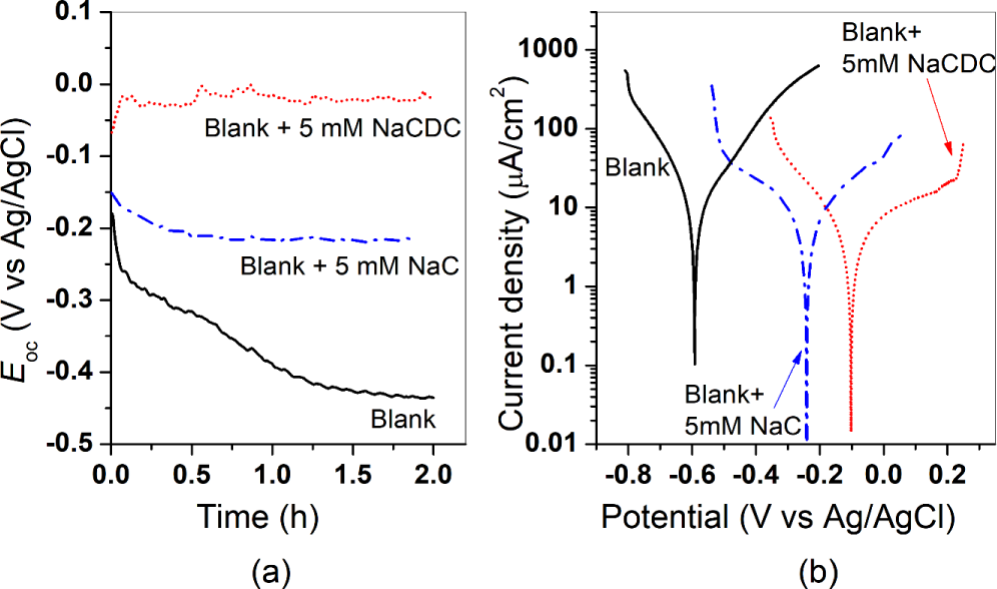
(a) Open-circuit potential variations vs time for carbon steel immersed in 0.02 M NaCl solution (blank) without and with additions of bile salts at a concentration of 5 mM. (b) Potentiodynamic polarization curves for the carbon steel electrode after 2 h immersion in the three solutions. Scan rate = 1 mV/s.

Figure 5a shows the evolution with time of *E*_oc_ for the carbon steel electrode in the blank solution (20 mM NaCl) without and with additions of 5 mM NaC and 5 mM NaCDC. The open-circuit potential for the sample immersed in the blank solution has an initial value around −200 mV vs Ag/AgCl. It exhibits a trend toward more negative values, reaching −450 mV after 2 h of immersion. Such a trend can be ascribed to the breakdown process of the native oxide film.^12^ It is well-known that a potential-pH diagram (Pourbaix diagram) enables prediction of the thermodynamic stability of iron in aqueous solutions. At pH 7.0, the active corrosion region (where Fe^2+^ is stable) is characterized by a potential range with a lower limit, ca., −600 mV vs Ag/AgCl.^8,12^ The *E*_oc_ of −450 mV attained by the steel sample after 2 h immersion in the blank solution is slightly above such a limit and consistent with the sample being in the active corrosion state.

The Pourbaix diagram also shows that the transition from the active to the passive region (region of stability of hydrated Fe^3+^ oxides that form dense protective films) occurs with a change in potential in the positive direction. More specifically, it has been shown in the literature that spontaneous passivation in open-circuit conditions (chemical passivation) of iron or mild steel in neutral aerated solutions occurs at potentials within the range of −100 to −150 mV (Ag/AgCl).^38,39^ The *E*_oc_ for carbon steel in the solution containing 5 mM NaCDC shows a shift toward the anodic direction from its initial value and stabilizes at −25 mV, giving a clear indication that the carbon steel surface becomes passivated (with formation of a protective iron oxide film) in the presence of chenodeoxycholate. On the other hand, the *E*_oc_ in the solution with the addition of 5 mM cholate exhibits a shift toward more negative values and rapidly attains a stable value of, ca., −200 mV. This potential is still within the active corrosion region but near the passive region. Potentiodynamic polarization measurements were carried out to get a better insight into the protective properties of the bile salts and their effect on the kinetics of the anodic and cathodic reactions.

Typical polarization curves obtained for carbon steel after 2 h of immersion in the blank and inhibited solutions are presented in Figure 5b. It is evident that in the presence of the bile salts, there is a shift of the corrosion potential (*E*_corr_) toward less negative values and, at the same time, a decrease of both cathodic and anodic current densities (in the order NaC < NaCDC). The shift of *E*_corr_ follows the same trend in *E*_oc_ values after 2 h immersion of the steel electrode in the blank and inhibited solutions, confirming that there is a tendency to change from the active to passive state in the presence of bile salts. The decrease in cathodic current densities in the solutions containing bile salts can be related to the progressive deposit of insoluble species which act as a barrier for oxygen diffusion. The decrease in anodic current densities, indicating a suppression of the iron dissolution reaction, involves the role of dissolved oxygen to convert Fe^2+^ ions to Fe^3+^ in local anodic areas and formation of weakly soluble ferric compounds.^9,14^ As discussed further below, the adsorption of cholate or chenodeoxycholate anions through the side chain of the COO^–^ group is crucial for passivation. However, the anodic branch of the polarization curves in Figure 5b gives evidence that only the chenodeoxycholate anion acted as an effective passivator (in agreement with *E*_oc_ values in Figure 5a), since only the polarization curve obtained in the solution with NaCDC exhibits a passivation plateau. Corrosion kinetic parameters were determined from these polarization curves by the Tafel extrapolation method, and the average results are presented in Table 1. The average corrosion current density (*i*_corr_) decreases from 8.5 μA/cm^2^ in the blank to 3.8 μA/cm^2^ in the blank solution with the addition of 5 mM NaC and to 1.7 μA/cm^2^ in the solution containing 5 mM NaCDC. The average inhibition efficiency  (presented in the last column in Table 1) was calculated according to , where  is the average corrosion current density in the blank solution and  is the average corrosion current density obtained in inhibited solution. Clearly, chenodeoxycholate acts as a better corrosion inhibitor (as indicated by a higher inhibition efficiency). Further information about the corrosion behavior of the steel electrode in the chloride solution containing additions of NaC and NaCDC was obtained from EIS measurements.

**Table 1:** Average Values for *E*_corr_ and *i*_corr_ Obtained by Tafel Extrapolation of Polarization Curves in Figure 5b (Standard Deviations Are in Parentheses) and Average Inhibition Efficiency  for Carbon Steel in Solutions Containing NaC or NaCDC

Electrolytes	*E*_corr_ (mV vs Ag/AgCl)	*i*_corr_ (μA cm^–2^)	(%)
Blank: 0.02 M NaCl, pH 7	–592 (3)	8.5 (2.3)	
Blank + 5 mM NaC	–254 (12)	3.8 (0.8)	55
Blank + 5 mM NaCDC	–90 (15)	1.7 (0.3)	80

**Figure 6:**
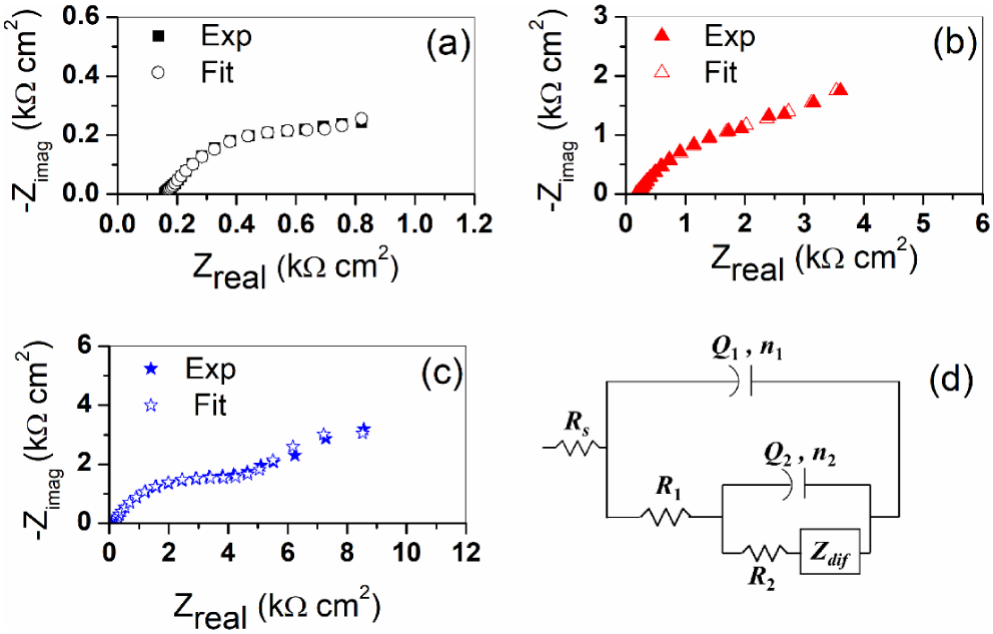
(a–c) Impedance spectra (Nyquist plots) for carbon steel electrodes after 2 h immersion in the blank, blank + 5 mM NaC, and blank + 5 mM NaCDC solutions, respectively. In each case, the solid symbols correspond to experimental data, while open symbols resemble the best fit with the equivalent circuit in (d).

Figures 6a–c show the typical Nyquist diagrams obtained for carbon steel after 2 h of immersion in the three solutions mentioned above. It is evident that the impedance magnitude increases in the solutions containing the inhibitor, and that in the three cases, the impedance response involves two-time constants. Thus, they were modeled with the equivalent circuit in Figure 6d, where *R*_*s*_ is the electrolyte resistance and *R*_1_ and *R*_2_ are the resistances associated with the high-frequency (HF) and low-frequency (LF) time constants, respectively. Although the two loops observed in the Nyquist diagrams are taken as capacitive semicircles, a constant phase element (CPE) was associated with each one (instead of a capacitance) in order to account for the deviation from ideal capacitive behavior, typically attributed to surface inhomogeneity.^40^ The admittance of the CPE is given by *Y*_CPE_ = *Q*(ω)^*n*^, where ω is the sine-wave modulation angular frequency, *Q* is the base admittance (with dimensions Ω^–1^ s*^n^* cm^–2^), and *n* is an empirical exponent (0 ≤ *n* ≤ 1), which measures such a deviation from ideality.

The equivalent circuit in Figure 6d represents an imperfectly covered electrode. The HF time constant (linked to *R*_1_ and *Q*_1_) can be attributed to the presence of a layer. In contrast, the LF time constant (linked to *R*_2_ and *Q*_2_) is related to uncovered areas where charge transfer (and possible diffusion) is involved in the corrosion process. The good agreement between the experimental and fitted data in Figure 6a–c indicates the excellent quality of the fittings with this model. The optimum fit parameters are summarized in Table 2. The base admittance *Q*_1_ is in the order of 1 mF s^(*n*1–1)^ cm^–2^ for the steel electrode immersed in the blank solution and blank + 5 mM cholate, while its value for the solution containing NaCDC is 145 μF s^(*n*1–1)^ cm^–2^. This observation allows us to interpret that the HF loop in Figure 6a,b corresponds to the response of a highly porous and inhomogeneous corrosion product layer, while the HF loop in Figure 6c can be ascribed to the impedance response of a protective organic layer. Furthermore, considering that *R*_2_ corresponds to the charge-transfer resistance *R*_ct_, the results of EIS measurements can also be used to evaluate the inhibition efficiency according to the formula η^*ac*^ = (1 − (*R*_ct_^*B*^)/(*R*_ct_^*I*^)) × 100, where the superscripts *B* and *I* refer to blank and inhibited solutions. The η^*ac*^ values agree with those in Table 1, obtained from polarization curves, confirming that chenodeoxycholate is a better corrosion inhibitor, compared to cholate, at the same concentration.

**Table 2:** Optimum Fit Parameters from Equivalent Circuit Analysis of the Impedance Spectra in Figures 6a–c^a^

	Electrolytes		
Parameters	Blank	Blank + 5 mM NaC	Blank + 5 mM NaCDC
*R*_s_ (Ω cm^2^)	159.1	176.1	182.8
*Q*_1_ (μF cm^–2^ s^(*n*1–1)^)	1104.2	994.5	145.5
*n*_1_	0.65	0.60	0.65
*R*_1_ (Ω cm^2^)	68.2	633.5	171.0
*Q*_2_ (μF cm^–2^ s^(*n*2–1)^)	936.9	100.98	13.31
*n*_2_	0.73	0.84	0.99
*R*_2_ (kΩ cm^2^)	0.57	2.93	4.30
σ (Ω cm^2^ s^–1/2^)	41.27	179.90	428.60
τ_diff_ (s)	79.17	-	124.50
η*^ac^* (%)		62	87

a Values of inhibition efficiency η^*ac*^ are also included.

Table 2 also shows values of parameters related to the diffusion impedance (Warburg coefficient σ and diffusion time constant). The value of σ represents the resistance of diffusion through the inhibitor layer. As discussed above, the cathodic branch of polarization curves (Figure 5b) gave an indication that the layer developed in the presence of bile salts acts as a barrier for oxygen diffusion. Such a behavior is confirmed here with the σ values which are higher for the steel sample that was immersed in the blank solution with additions of NaC or NaCDC.

NaC is a trihydroxy bile salt with hydroxyl groups at positions and orientation 3α, 7α, and 12α, while NaCDC has only two hydroxyl groups at 3α and 7α. The results of electrochemical measurements suggest that this structural difference between these two bile salts has a determining effect on the self-assembly process and the corrosion inhibition mechanism (as discussed further below). To elucidate the interaction between cholate and chenodeoxycholate with the steel surface, the composition of surface films was investigated by using XPS.

### X-ray Photoelectron Spectroscopy Analysis

**Figure 7:**
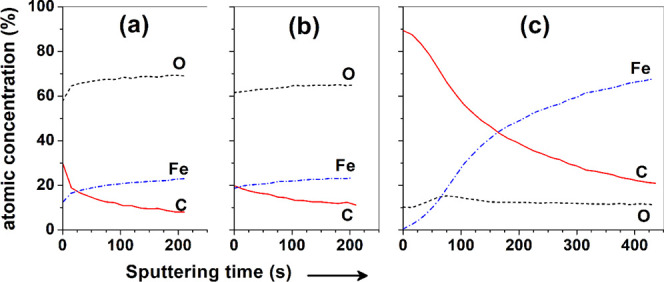
Comparison of XPS depth concentration profiles for surface films formed on carbon steel after 2 h immersion in (a) blank, (b) blank + 5 mM NaC, and (c) blank + 5 mM NaCDC solutions.

**Figure 8:**
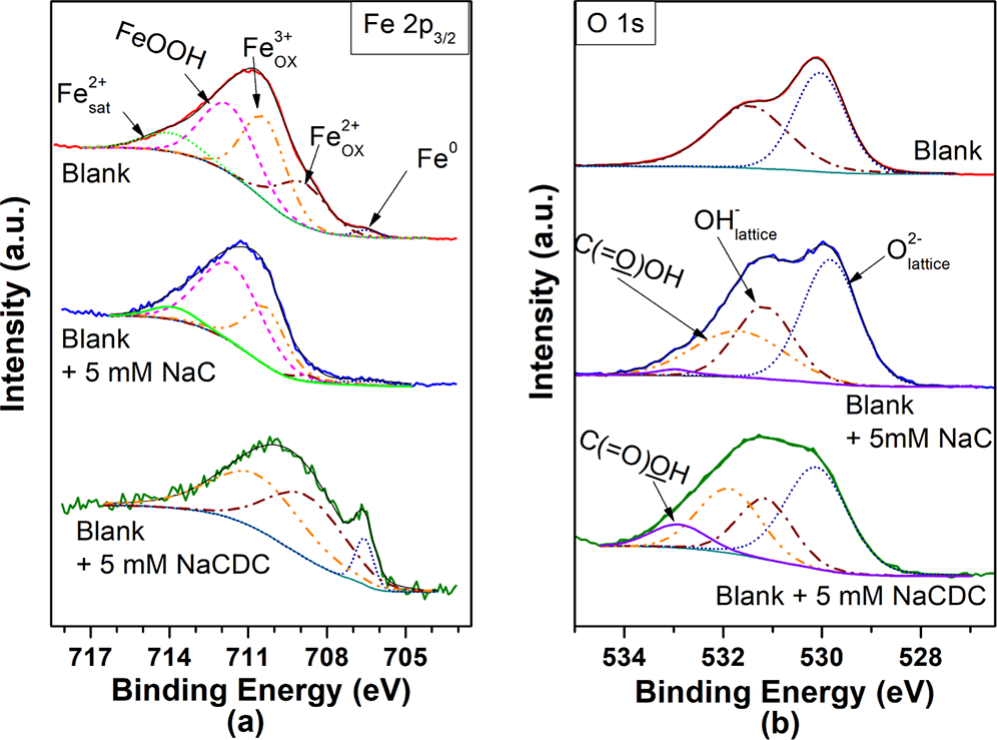
High-resolution XPS spectra of the (a) Fe 2p_3/2_ and (b) O 1s regions were obtained for surface films formed on carbon steel after 2 h of immersion in the blank and inhibited solutions. Their deconvolutions are also displayed.

Figure 7 presents the XPS depth concentration profiles for samples that were exposed for 2 h in blank and inhibited solutions. The outermost concentration of C for the steel sample exposed to the blank solution (Figure 7a) is 30 at. % and drops rapidly with sputtering time, indicating that it is mainly due to carbonaceous contamination. For the sample that was immersed in the solution with inhibitor NaC, the concentration of C at the topmost surface is only 20 at. % (Figure 7b), decreases slowly with etching, and levels off at a slightly higher value than that observed in Figure 7a. As confirmed later with deconvolution of the O 1s spectra in Figure 8b, this depth distribution of the carbon atom can be attributed to the adsorption of a certain amount of cholate ions. The depth distributions of Fe and O atoms reveal the presence of a thick rust layer on the surface of steel immersed in both, the blank solution and the one containing NaC. A surface O/Fe atom ratio >2, estimated from the profiles in Figure 7a,b, suggests that the rust layers contain Fe(OH)_2_ (and possibly FeOOH) in both cases. The elemental depth profile for the sample immersed in the solution with additions of chenodoxycholate (Figure 7c) is entirely different. A very high concentration of C is observed at the topmost surface, which decreases gradually with sputtering time, reaching a value of 20 at. % after 400 s. Such a depth distribution of C clearly indicates the presence of an organic layer: chenodeoxycholate molecules self-assembled in the steel surface. Thus, the elemental depth profiles presented in Figure 7 confirm the results of electrochemical impedance measurements in the sense that the high inhibition efficiency in the presence of chenodeoxycholate is related to the formation of a protective organic layer.

To get quantitative insights into the surface composition of the films formed on the steel immersed in the three solutions, peak fitting of high-resolution spectra for Fe 2p_3/2_ and O 1s was carried out. Figure 8 shows the high-resolution Fe 2p_3/2_ and O 1s spectra obtained at a takeoff angle of 0° for carbon steel samples that were immersed during 2 h in the blank solution and in blank solutions with additions of the bile salts. A deconvolution of the Fe 2p_3/2_ spectra in Figure 8a was carried out, and the results show that each spectrum consists at least of 3 subpeaks, corresponding to Fe^0^, Fe^2+^, and Fe^3+^. Two additional subpeaks are observed (attributed to FeOOH and a satellite peak) only in the Fe 2p_3/2_ peaks obtained for steel immersed in the blank and in the blank + 5 mM NaC solutions. The binding energies for all subpeaks are in agreement with literature values.^41,42^

Deconvolution of the O 1s spectra in Figure 8b shows the presence, in all of them, of two broad subpeaks characteristic of oxygen in oxide (O^2–^) and hydroxide (OH^–^) environments. Furthermore, the spectra corresponding to specimens that were immersed in solutions containing cholate or chenodeocycholate exhibit two additional subpeaks at higher binding energies, which can be attributed to carbonyl oxygen (C(=O) OH) and noncarbonyl oxygen in carboxyls (C(=O) OH).^43,44^ The peak fittings for the carbon steel sample immersed in the blank solution suggest that the layer of corrosion products consists mainly of iron oxides (FeO and possibly Fe_3_O_4_) and hydroxides (Fe(OH)_2_ or Fe(OH)_3_), with some FeOOH also present. For carbon steel immersed in solution with the addition of 5 mM NaC, the layer is composed predominantly of FeOOH and Fe(OH)_3_. The contribution of ferrous ions is lower than that observed in the layer of corrosion products developed in the blank solution (consistent with a lower corrosion rate). The fact that the O 1s peak exhibits the presence of carbonyl oxygen suggests that cholate ions did adsorb on the metallic surface (and, according to the results of electrochemical measurements, promoted a modest corrosion inhibition effect). It is interesting to observe that the metallic iron signal in the Fe 2p_3/2_ spectra is very small for the samples immersed in the blank and blank + 5 mM NaC solutions due to a thick layer of corrosion products; however, it is significant in the sample immersed in a solution containing NaCDC. This means the protective layer developed in such a solution involves a thin passive film composed (most likely) of Fe_3_O_4_ and FeOOH.^35^ Furthermore, Figure 8b shows a significant contribution of carbonyl oxygen and noncarbonyl oxygen in its O 1s peak, clearly associated with the organic layer developed on the carbon steel surface immersed in the solution containing chenodeoxycholate. This is supported by the analysis presented below for high-resolution C 1s spectra obtained with ARXPS.

**Figure 9:**
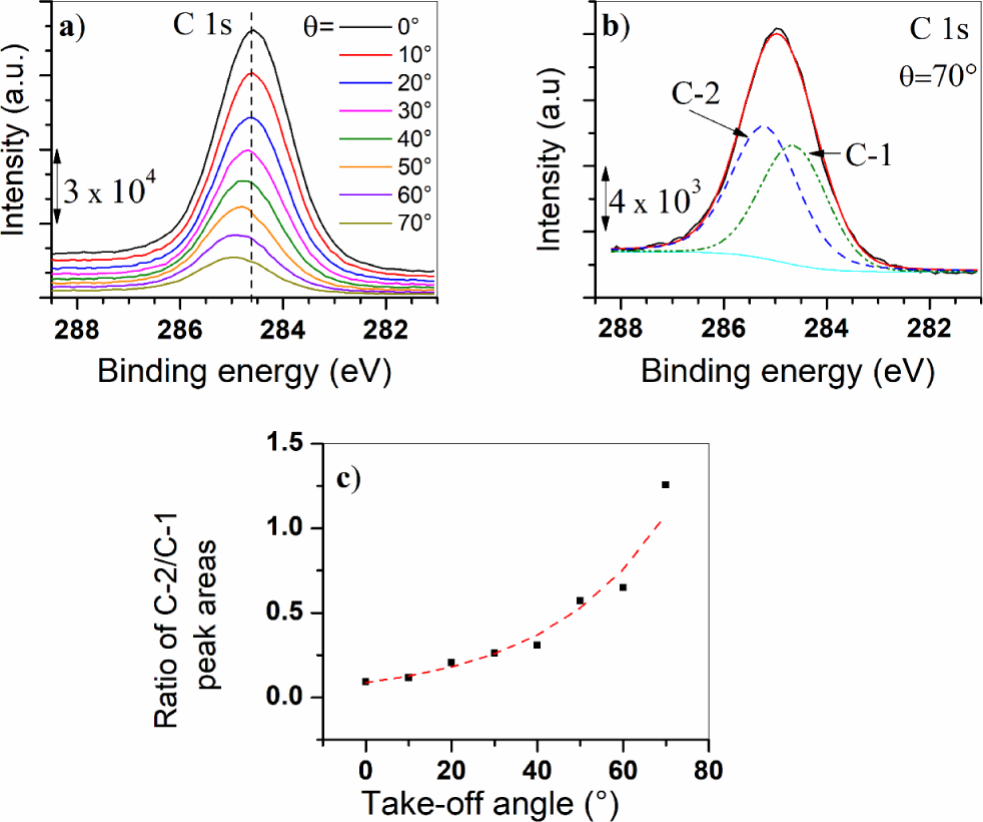
(a) C 1s angle-resolved XPS spectra for carbon steel immersed during 2 h in the solution containing NaCDC, (b) deconvolution of the high-resolution C 1s spectrum obtained at an angle of 70° (the interpretation of subpeaks C-1 and C-2 is explained in the text), and (c) ratio of C-2/C-1 peak areas as a function of takeoff angle.

Figure 9a shows the C 1s spectra obtained as a function of takeoff angle (between 0° and 70°) for the carbon steel sample immersed in the solution containing chenodeoxycholate. At an angle of 0°, the excitation signal reaches the bulk of the film and the peak intensity is high. As the takeoff angle increases, the intensity decreases, but surface sensitivity improves, and the composition in the outer part of the film becomes important. The results of deconvolutions showed that actually, each C 1s spectrum consists of two subpeaks located at ∼284.5 and 285.1 eV and ascribed, respectively, to C–C/C–H structural features and to the carbon atom attached to carboxylic carbon (C-COOX).^45,46^ As an example, the deconvolution for the C 1s peak corresponding to an angle of 70° is presented in Figure 9b, where the two subpeaks were labeled as C-1 and C-2. Although these features are often related to an overlay of carbonaceous contamination, there is an interesting observation that suggests that, in fact, the signal originates from the outermost region of the organic layer, already detected with XPS depth concentration profiles (Figure 7c): concomitant with the decrease of intensity (as the takeoff angle increases), there is a slight shift of the C 1s peak maximum toward higher binding energies. This can be observed in Figure 9a, which takes the dashed vertical line as a reference and can be interpreted as an increase in the dominance of peak C-2 for higher takeoff angles, that is, the dominance of peak C-2 (corresponding to C-COOX) in the outer region of the organic layer. To quantify this observation, Figure 9c shows the ratio of C-2 to C-1 peak area as a function of the takeoff angle of photoelectrons. This graph suggests that the side chain of the chenodeoxycholate molecule is oriented outward. In other words, it provides some insights into the self-assembly of this bile salt on the carbon steel surface, as further discussed below.

### Self-Assembly of the Bile Salts at the Surface of the Carbon Steel and Possible Corrosion Inhibition Mechanism

Molecular self-assembly is an autonomous process in which molecules (such as surfactants, peptide amphiphiles, dendrimers, etc.) gather to form organized structures. It is mediated by weak, noncovalent interactions like hydrogen bonding, van der Waals interactions, hydrophobic interactions, etc.^45^

Since the bile salts are amphipathic molecules, they can form micelles; however, the self-assembly mechanism cannot be predicted according to the rules for conventional surfactants,^46^ because of their rigid structure and facial amphiphilicity. Although different aggregation mechanisms have been proposed in the literature, there is a general agreement that aggregation of bile salts is driven by the interplay of hydrophobic interactions and hydrogen bonds involving hydroxyl and carboxylic groups.^34,47^ For instance, the earliest model involves a stepwise mechanism, wherein in the first step, the bile salts hydrophobically associate with their hydrophobic faces turned to each other (expelling water from the interior of the aggregate), forming a globular “primary micelle”, while in a second step, at higher concentration, the hydroxyl groups of different primary micelles interact through hydrogen bonds, leading to the formation of elongated “secondary micelles”. Other possible micellar structures are the disklike or helical micelles.^34^

As indicated above, NaC contains three hydroxyl groups, while NaCDC has only two. Such a difference in the number of hydroxyl groups is expected to affect their self-assembly behavior. In fact, the cholate ion is characterized by the most pronounced biplanarity given the hydrophobic β-face and hydrophilic α-face; however, in the case of the chenodeoxycholate ion, the polar groups are arranged along one edge of the tetracyclic ring system. Thus, this ion is characterized by a hydrophilic edge (rather than a hydrophilic face). Computational studies have been conducted by different authors to contribute to the understanding of micelle formation from bile salts. For instance, Pártay et al.^48,49^ used molecular dynamics simulations at an atomistic level to show that neighboring cholate ions prefer to aggregate as face-to-face type arrangements (kept together either by hydrogen bonding or by hydrophobic interactions), while for deoxycholate (a dihydroxy bile salt), pairs of ions appear to be stuck together by hydrogen bonds at the edges, thus allowing the opening of the angle between the planes of the two tetracyclic ring systems. Haustein et al.^50^ also studied the formation of micelles from cholate ions. Their results of Brownian dynamics simulations for a coarse-grained model agreed with the stepwise mechanism, indicating that cholate molecules are preferentially arranged in a back-to-back manner with their hydrophobic faces turned to each other.

**Figure 10:**
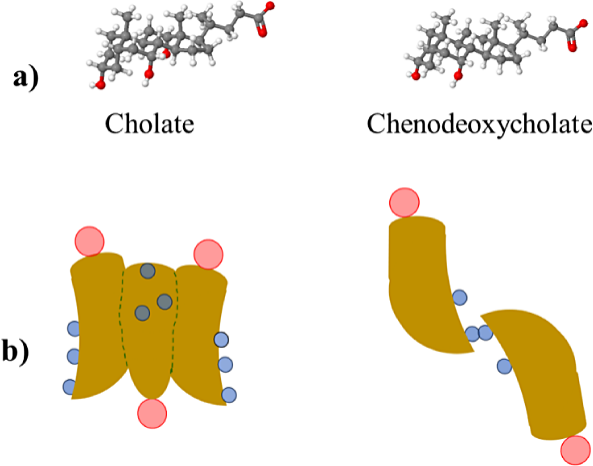
(a) Ball and stick representation of the structure of cholate and chenodeoxycholate anions and (b) schematic representation of primary micelles formed with cholate and chenodeoxycholate anions (forming a trimer and dimer, respectively). In the graphic representation of each bile salt anion, the small blue circles represent the hydroxyl groups and the pink circle represents the carboxylate group (COO^–^).

Furthermore, cluster size distributions at ambient temperature for cholate concentrations between 1 mM and 150 mM showed that micelles with aggregation numbers 3 and 4 occur with the largest probability.^50^ The results indicated that for a concentration of 3.5 mM (near to that used in this work), trimers are more likely to form. According to these theoretical studies, it would be reasonable to assume that for the concentration of 5 mM, cholate ions are self-assembled in the aqueous solution, forming a trimer through hydrophobic interactions, and chenodeoxycholate ions form a dimer stuck together by one hydrogen bond. Figure 10a shows the ball and stick representations of cholate and chenodeoxycholate ions, and Figure 10b shows schematic representations of the primary micelles formed with cholate and chenodeoxycholate ions.

**Figure 11:**
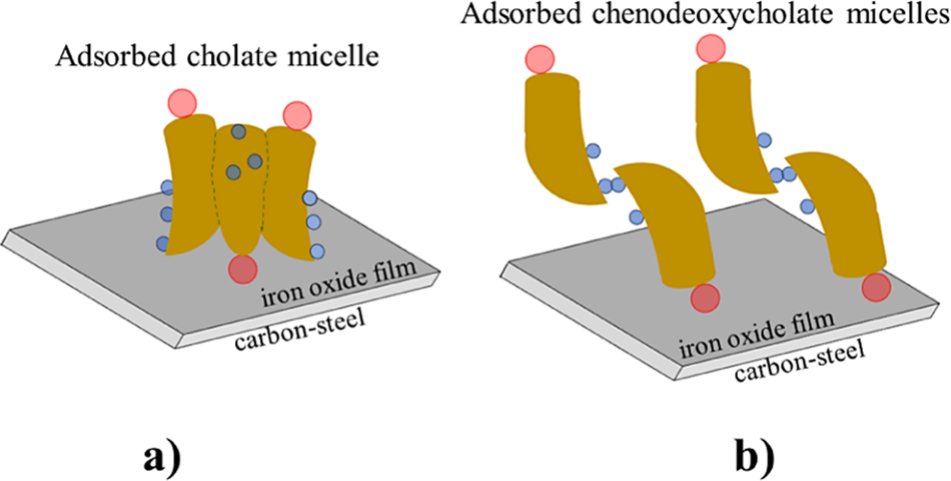
Schematic representation of the adsorption of (a) cholate micelle and (b) chenodeoxycholate micelles on the hydroxylated iron oxide surface. In the graphic representation of each bile salt anion, the small blue circles represent the hydroxyl groups, while the pink circle represents the carboxylate group (COO^–^).

The primary concern for a biosurfactant to act as a corrosion inhibitor is its ability to be adsorbed on the metal surface. An important fact to consider (with regard to the adsorptive mechanism) is that an air-formed oxide film covered the carbon steel electrode used in this work. As reported elsewhere, the isoelectric point (iep) for such a native oxide film is typically in the range of 8–10.^51,52^ Thus, in an aqueous solution with pH 7, the adsorption process would involve an interaction between the cholate or chenodeoxycholate micelles and a positively charged hydroxylated oxide surface. Two possible configurations can be envisioned: one in which the primary micelles are lying down (with hydroxyl groups in the α-face or methyl groups in the β-face interacting with surface OH groups) and another in which the micelles stand up with the carboxylate in the side chain acting as the anchor group. The stand-up configuration (schematized in Figure 11) is the most plausible since the electrostatic attraction between COO^–^ and the positive surface is stronger than the van der Waals interactions. This adsorption mechanism resembles closely that of straight-chain aliphatic carboxylates^14,15^ (the inhibitor is bonded by the carboxylate group to the hydroxylated iron oxide surface). Thus, it seems interesting to compare the inhibition efficiencies obtained in this work with those corresponding to straight-chain saturated monocarboxylates or dicarboxylates under similar experimental conditions. For instance, for a monocarboxylate with 5 carbon atoms (sodium valerate) at a concentration of 5 mM, Hefter et al.^7^ reported a corrosion inhibition efficiency of 78% for mild steel in a 9 mM NaCl solution. Similarly, for a dicarboxylate with 5 carbons (sodium glutarate) at a concentration of 5 mM, Chan-Rosado and Pech-Canul^53^ obtained an efficiency of 64% for carbon steel in a neutral 20 mM NaCl solution. Accordingly, the inhibition efficiencies of NaC and NaCDC are in reasonable agreement with those of conventional aliphatic carboxylates.

Our XPS measurements show evidence that in the solution containing cholate ions, the carbon steel surface is covered mainly by corrosion products (i.e., the corrosion rate decreased slightly in the presence of 5 mM NaC) with cholate molecules adsorbed only at some surface sites. On the other hand, chenodeoxycholate ions self-assembled at the interface form an organic layer (sealing defects in the native oxide film) and, at the same time, promote the formation of a protective iron passive film (the reduction of dissolved oxygen leads to the conversion of Fe^2+^(ac) to the ferric state). Such corrosion and corrosion inhibition behavior can be explained considering the adsorption mechanism proposed above. For cholate, the globular primary micelle (Figure 11a) occupies a large surface area, but only one anchor group points to the surface. This suggests that the carboxylate group binds to the steel surface only at a few sites, leaving most active sites exposed to the corrosive medium. Conversely, the open structure of the chenodeoxycholate dimer (Figure 11b) allows binding of more anchor groups to the carbon steel surface. Such a configuration becomes advantageous for the formation of a densely packed organic layer. This agrees with the ARXPS data analysis for C 1s, which suggests (Figure 9c) that in the outer region of the organic layer, the side chain is oriented toward the bulk solution.

## Conclusions

The protective effect of cholic and chenodeoxycholic acid sodium salts against the corrosion of carbon steel in a neutral 20 mM NaCl solution was investigated by using potentiodynamic polarization and electrochemical impedance measurements. The results indicate that when added at a concentration of 5 mM, both bile salts act as corrosion inhibitors, although they do not have the same protection ability. NaCDC exhibits the best performance (achieving a corrosion inhibition efficiency ≈85%). The composition of surface films grown on carbon steel by immersion in the corrosive and inhibited solutions was investigated with XPS. It was found that in the solution containing NaC, a layer of oxidic corrosion products was formed on the steel surface (due to poor corrosion inhibition), while in the presence of NaCDC, the adsorption process led to the formation of an organic layer that promoted the passivation of steel. The fact that these two inhibitors did not exhibit the same protective efficiency was attributed to differences in their self-assembly behavior, given their structure (which led to the formation of a densely packed organic layer in the case of NaCDC, but not for NaC). It has been hypothesized, based on theoretical calculations reported by other authors, that at the concentration used in this work, cholate ions form trimers by hydrophobic interactions and chenodeoxycholate ions form dimers through hydrogen bonds at the edges. In both cases, the carboxylate group in the side chain is assumed to act as the anchor group, which points to the surface. It was proposed that adsorption on the steel surface occurs through electrostatic interactions between the side-chain COO^–^ group and the positively charged oxide surface. This adsorption configuration was confirmed by angle-resolved XPS measurements for the steel sample treated with NaCDC.
